# The Use of Repertories in Finding the Homœopathic Remedy

**Published:** 1892-02

**Authors:** Horace P. Holmes

**Affiliations:** Omaha, Nebraska


### THE USE OF REPERTORIES IN FINDING THE HOMŒOPATHIC REMEDY.

HORACE P. HOLMES, M. D., OMAHA, NEBRASKA.

Read before the Omaha Homœopathic Medical Society.

Hahnemann says in Par. 104 of the *Organon:* " When all of the prominent and characteristic symptoms, collectively forming an image of a case of chronic, or of any other disease, have been carefully committed to writing, the most difficult part of the labor will have been accomplished." Again, in Par. 147, he says: " A drug, completely tested with regard to its power of altering human health, and whose symptoms present the greatest degree of similitude with the totality of symptoms of a given natural disease, will be the most suitable and reliable homœopathic remedy for that disease, for which the specific curative agent will have been discovered."

Accepting these statements as literally true, we find the next question confronting us a serious one. *How to find the remedy?* If our materia medica were so small that every symptom could be committed to memory and the whole field of materia medica form a picture, or a series of pictures, which could be readily called to mind, the task would be an easy one. But such is far from the case. Our remedies are numbered by the thousand and our symptomatology has grown to such gigantic proportions that it easily fills ten large octavo volumes, and the end is not yet. In the face of this great mass of material the question " How to find the remedy?" is often discouraging and sometimes hopeless. The practice of memorizing " key-notes " is of great service in the bedside practice, and may give a capacity sufficient to allow the practitioner to treat the ordinary cases that come to him. But when a patient seems to present " key-notes " of almost every remedy we can think of, or when these " key-notes " prove to be symptoms common to several remedies, then the searcher for the simillimum finds the task too great an one to be solved by the aid of memory alone.

It is the solution of such cases that gives help to our fellow-workers, and each man's method may be a help to us all. That a case properly taken can be prescribed for and the true simillimum found, is almost as certain as that a problem in mathematics can be solved if the rule is given.

When we reach the end of memorizing, then we must use keys in the study of materia medica. The more repertories one has the better is he prepared to search for the true remedy. There is thus far no *best* repertory, as each one must possess advantages peculiar to the individual and the composite group makes the perfect set. Therefore, the more repertories the better.

As a practical illustration of " How to use a repertory," I will recite an actual case in practice and give the details of each step in the search for the remedy. I do not claim to be the originator of the method, although I know of no one that used it before myself. The only claim I make for it is that it is the best scheme I know of for finding the exact remedy in puzzling cases. That it can be improved upon there is no doubt, and practice and familiarity with the work will reveal many ways of shortening it. In my illustration I use the entire repertorial rubrical lists in order that no one may be puzzled by an attempt to show the shorter cuts.

I will report the following :

### A Case of Sick-Headache.

January 24th, 1891.

Mr. ——— aged fifty, a shipping clerk, was directed to come to me in order to get relieved from his frequent attacks of sick-headache. I drew out the following history and picture of the case. When a school-boy the patient would have to go home from school on account of violent sick-headache. Was compelled to go to bed from the severe pain and prostration. The attacks came as often as every two or three weeks. This has been the case all his life, until of late the attacks have been more frequent, now coming on every week, or twice a week. Both his father and mother were subject to frequent attacks of sick-headache.

The typical headache came with a pain in the left temple ; there was nausea and gagging, but no vomiting. Came on during the day, and he would have to go home and lie down, and be perfectly quiet. By the next morning he would be all right, as a night's rest generally relieved the whole trouble.

At the present time the headache is more severe. He gets up in the morning with a dull feeling in the vertex along the sinus, is chilly. By afternoon the headache is so bad that he cannot think nor reason. The pain passes down to left base of occiput, prostrates him, and he has to give up work and go home. The pains are darting, shooting up into vertex along sinus, and cut like a knife. Sensation as if the top of the head would split open. He has to sit up with head leaning backward. The farther the head can be thrown backward the more relief he gets. Bandaging the head controls the pain somewhat. Feet get cold to the knees, but are not damp. The headache is always aggravated by the feet getting cold. There is now no nausea. Appetite good even while suffering great pain, and as a rule eating brings relief from the pain, but does not cure. Puts the feet in hot water, and as hot cloths as can be wrung out of water with the hands are put to the head, and the heat brings some relief. Must keep the head hotly bandaged or covered. The least cold aggravates. The cloths cooling, an open door or draft of air makes the pain worse. When he gets thoroughlv warmed through, he goes to sleep, and wakes up all right in the morning.

The attacks are brought on by taking cold, by getting cold, or by getting the feet very cold ; by over-work, either mental or muscular.

Concomitants: He is cross and irritable with the pain ; the bowels are regular ; no thirst ; pain in kidneys, worse on left side ; a steady pain over the kidneys, worse lifting or from much walking ; itching of the anus, worse undressing, and relieved by hard scratching.

Aggravations: From noise, thinking, lying down, cold, motion, stooping, cold drafts. He dreads cold drinks.

Ameliorations: From quiet, sitting up, with the head thrown far back, very hot applications, wrapping up warmly, general warmth, bandaging, eating, hot drinks, sleep.

A rectal examination to search for the cause of the pruritus ani shows three irritable papillæ, deep, fissure-like depressions, and a few small internal piles.

I began the search for the remedy, which seemed very indefinite, by arranging the symptoms in the following rubrics:

1. Headache in the morning.
2. Pain going from vertex to occiput.
3. Shooting pains.
4. Splitting [bursting] sensation in vertex.
5. Great weakness.
6. Aggravation from lying down.
7. " " cold.
8. " " thinking.
9. " " stooping.
10. Ameliorated by hot applications.
11. " on awakening.
12. " from eating.
13. " from sleep.
14. " by bandaging.
15. " on wrapping up.
16. " from bending head backward.

---

Arranging the remedies as indicated under the rubrics we find them as follow:

*Headache in the morning.* Acon., Agar., All-s., Alum., Ambr., Am-c., Am-m., Anac., Ang., Ant-t., Arg., Arg-n., Arn., Ars., Asaf., Asar., Aur., Bar., Bell., Benz-ac., Berb., Borax, Bry., Bov., Cadm., Calc., Calc-ph., Camph., Cann-s., Canth., Carb-an., Carb-v., Caus., Cham., Chin., Chin-s., Cic., Cina, Clem., Coca., Coff., Coloc., Con., Creos., Croc., Croton., Cuprum., Cyc., Dios., Dulc., Eup-pur., Euph., Ferr., Glon., Graph., Grat., Guac., Hell., Hepar, Hipp., Ign., Indm., Iod., Ipec., Iris., Jatr., Ka-bi., Ka-c., Ka-iod., Kalm., Lac-c., Lach., Lachn., Led., Lil-t., Mag-c., Mag-m., Mang., Merc., Merc-j., Merc-s., Mezer., Murx., Mur-ac., Nat-c., Nat-mur., Nit-ac., Nitr., Nux-j., Nux-m., Nux-v., Ol-an., Paeon., Pau-p., Petr., Phos., Phos-ac., Phyt., Podo., Psor., Puls., Ran-b.,

Rheum, Rhod., Rhus, Rumx., Ruta, Samb., Sang., Sars., Seneg., Sep., Sil., Spig., Squil., Stann., Staph., Stram., Stront., Sul., Sul-ac., Tabac., Thuj., Verat, Zinc.

*Pain going from vertex to occiput.* Calc-ph., Chel., Gels.

*Pains shooting.* Acet-ac., Agar., Alum., Ambr., Am-c., Ant-t., Arg., Bar., Bell., Bry., Calc., Caps., Carb-v., Caust., Cham., Cimic., Colch., Con., Corn., Ferr., Gran., Hell., Hepar, Hura., Hyos., Ign., Indg., Iod., Ipec., Ka-c., Lach., Lact., Laur., Mag-c., Manc., Marum., Merc., Mur-ac., Naja., Nat-m., Nit-ac., Nitr., Nux-v., Petr., Plan., Ptel., Puls., Rhus-r., Sep., Sil., Staph., Sulph., Thuj., Tong., Valer.

*Splitting [bursting] pains in vertex.* Amm-m., Bapt., Calc., Carb-an., Cimic., Graph., Lac-ac., Nat-s., Nit-ac., Sil., Spig., Spong., Stront., Xanth.

*Great weakness [prostration].* Agar., Alum., Ant-c., Arn., Ars., Bov., Chin., Chin-s., Creos., Cup-ac., Cyc., Glon., Grat., Hydra., Hydr-ac., Hyos., Iod., Iris., Lil-t., Lobel., Mag-m., Nat-m., Nitr., Nux-v., Ol-an.. Phos., Psor., Ran-b., Rhus-r., Sil., Sul., Tarent.

*Aggravation from lying down.* Ambr., Amm-m., Arg., Ars., Asaf., Aur., Benz-ac., Cadm., Calc., Carbo-v., Caps., Caust., Cham,, Cist., Con., Cycl., Dig., Dros., Dulc., Euph., Euphr., Ferr., Hyos., Ign., Lith., Lyc., Mag-m., Meny., Mosch., Mur-ac., Nitr-ac., Nux-m., Nux, Phos-ac., Plat., Puls., Rhod., Rhus, Ruta, Sabad., Samb., Sep., Sil., Stront., Sulph., Tar., Thuj., Valer., Verb., Viol-tr.

*Aggravation from cold.* Acon., Agar., Alum., Amm-c.. Apis, Ars., Aur., Bar-c., Bell., Bry., Calc., Camph., Caps., Carb-an., Carb-v., Caust., Chin., Cist., Colch., Con., Daph., Dulc., Hell., Hep., Ign., Kali-b., Kali-c., Lyc., Mang., Merc., Mez., Mosch., Nux-m., Nux, Petr., Phos., Rhod., Rhus, Sabad., Sep., Sil., Stront., Verat.

*Aggravation from thinking.* Acon., Agn., Ambr., Amm-c., Anac., Aran-d., Arg-n., Arn., Asar., Aur., Bell., Cadm., Calc., Cham., Chin., Cimic., Cina, Cinnb., Cocc., Coff., Colch., Con., Daph., Dig., Fago., Graph., Hell., Hipp., Ign., Lach., Lact., Lyc., Mag-c., Nat-c., Natr-m., Nat-s., Nit-ac., Nux-m., Nux-v.,

Olnd., Op., Ox-ac., Par., Petr., Phos., Phos-ac., Plat., Psor., Ptel., Puls., Sabad., Selen., Sep., Sil., Staph., Stram., Sulph., Zinc.

*Aggravation from stooping.* Acet-ac., Acon., Æsc-h., Aloe., Alum., Amm-m., Ang., Ant-t., Apis, Arg., Arn., Asar., Bapt., Bar., Bell., Berb., Borax., Bov., Bry., Calc., Calc-ph., Camph., Canth., Caps., Carb-an., Carb-v., Caust., Cham., Chel., Chin., Chin-s., Cic., Cob., Cocc., Coff., Colch., Coloc., Con., Corn., Creos., Cupr., Cyc., Dig., Dros., Dulc., Ferr., Ferr-i., Form., Gels., Glon., Ham., Hell., Helon., Hepar, Hydrs., Hydr-ac., Hydrph., Hyos., Ign., Kali-b., Kali-c., Lach., Laur., Lyc., Mag-m., Manc., Mang., Marum., Men., Merc., Merc-i., Mill., Mur-ac., Nat-c., Nat-m., Nicc., Nitr., Nux-m., Nux-v., Par., Petr., Phos., Phys., Phyt., Pic-ac., Plat., Plect., Puls., Rheum, Rhus, Rhus-r., Samb., Sang., Seneg., Senn., Sep., Sil., Spig., Spong., Stann., Staph., Sulph., Sul-ac., Thuj., Valer., Verat., Vibur., Zing.

*Amelioration from hot applications.* Aur., Bell., Caps., Caust., Cinnb., Cocc., Colch., Kali-c., Kali-j., Lach., Mag-m., Nux-m., Nux-v., Rhod., Rhus, Sil., Staph., Stront., Sulph., Sumb.

*Amelioration on awakening.* [After sleep.] Acon., Bad., Glon., Hell., Hyos., Ign., Pallad., Sep., Sil., Thuj.

*Ameliorated by eating.* Argn-n., Arum-t., Carb-ac., Caust., Cist., Coca., Con., Gels., Gen., Indm., Lachn., Lyc., Petr., Phos., Phyt., Scut., Sep., Tellur., Tong.

*Ameliorated by bandaging.* Arg-n., Bell., Bry., Calc., Hepar, Mag-m., Nux-v., Psor., Puls., Rhod., Sil.

*Ameliorated by wrapping up.* Aur., Hepar, Mag-m., Mur-ac., Nux-v., Psor., Sil.

*Ameliorated by bending the head back.* Apis, Glon., Phos-ac., Thuja.

I have a "Repertory Checking List" which consists of the abbreviations of the remedies found in Lippe's *Repertory* arranged in columns like a sheet of labels. With a page of this checking list before me, I pass rapidly over the remedies found in the repertories under each rubric and check after each remedy on the checking list. Passing through the sixteen rubrics I find the valuation of the remedies standing highest was as follows: Silicea 12; Nux-vomica 10; Calcarea-carb. 8, and Sepia 8.

Although I had several remedies in my mind, this examination showed Silicea so far ahead that on further studying the materia medica I became convinced that I had found the remedy. At the next visit of my patient I questioned him further with the view of confirming or not the indication of Silicea. The following additional symptons of the remedy were brought out in confirmation of the correctness of the work: Intense religious emotion or listening to deep sermons or lectures brings on the headaches. There is often a vertigo with a whirling sensation; has had falling to the right or forward. Has fetid foot-sweat—must wash the feet every day in summer to keep the feet from smelling foul. Blisters on the little toes and between the toes when on the feet much. Trembling and weakness of the legs—it makes him nervous. Profuse axillary sweat—drops away, bad smelling.

There was now no doubt in my mind as to the remedy, and I gave him Silicea 1000th, two doses to be taken an hour apart, and a vial of placebo, and asked him to report in a week. He seemed to gain in a general way from the very start. In a few days, however, he came to my office saying he was in for one of his "old terrors." That his head felt as it always did when he was going to have the worst form of attack. He begged me to relieve him so that he would not have to go home. With much misgiving I gave him one dose of the 200th and a placebo "strong," and he went back to his store and missed his attack of headache for the first time almost in his life. Twice afterward he received a single dose of the 1000th and nothing more. Nor has there been any other medicine given him for the headaches since.

I saw the patient to-day (November 19th, 1891) and he said he had not had a headache since I prescribed for him. He passed through the hot summer without having any trouble from sweaty feet and the axillary sweat bears the same report. Thus far he has not suffered from the cold weather of this fall. Always before he would be subject to attacks of sick-headaches from getting his feet cold while attending to the transaction of his business. He is a better man physically and can stand up under more pressure and attend to more business than for a long time. Silicea touched the weak spot and it has made him a strong man, not only by its relieving him of the frequent attacks of sick-headaches, but of the many other symptoms complained of.

Such is an illustration of the work that may be done with the thorough use of repertories. In reply to the statement usually made in regard to such work that "it takes too much time," let me say No! It is true that it took me some time in the beginning—perhaps an hour and a half—I was not so familiar with the method then, as I can now unravel a similar case in twenty minutes after I have taken the notes. But the saving of time comes in the after-treatment. There is no farther study to be done so long as the remedy is indicated, and when that indication ceases we know at once what remedy stands next highest on the list.

